# Social media integration in medical training: behavioral impact of short-form video creation as an active learning tool

**DOI:** 10.3389/fmed.2025.1666255

**Published:** 2025-10-29

**Authors:** Lourdes De la Peña-Fernández, Irene Zapata-Martínez

**Affiliations:** ^1^Department of Radiology, School of Medicine, University of Málaga, Málaga, Spain; ^2^Department of Pharmacology and Pediatrics, School of Medicine, University of Málaga, Málaga, Spain

**Keywords:** medical education, students’ behavior, technology in medical sciences, motivation, active learning, social media

## Abstract

**Introduction:**

The rapid advancement of technology has transformed student behavior, increasing the demand for active learning strategies that integrate digital tools into medical education. Short-form video platforms such as TikTok offer an attractive format to enhance motivation and engagement among digital-native students. This study aimed to analyze the use of student-created videos in Radiation Oncology, evaluating their characteristics and, above all, students’ perceptions regarding their usefulness and their impact on competencies relevant to clinical practice.

**Methods:**

A cross-sectional study was conducted with 166 medical students at the University of Malaga. Of these, 148 (89.2%) voluntarily created short educational videos on course content. Student perceptions were measured through a validated 17-item questionnaire. Data were examined through a mixed-methods approach integrating quantitative and qualitative analyses.

**Results:**

A total of 24 videos were produced. Nineteen videos (79.2%) were uploaded to TikTok. The questionnaire was completed by 144 students (97.3%): 91% expressed enthusiasm for innovative methodologies, 86% reported improved content assimilation, and 87% highlighted enhanced memory retention. Correlational analysis showed positive associations between interest in innovative methods and perceived research skills, memory retention, and satisfaction. Preference for traditional methods correlated negatively with memory retention and innovation interest. Teamwork (57%) and creativity were the most valued benefits; while editing and organizational demands posed the main challenges.

**Conclusion:**

Student-created short videos, particularly via TikTok, were well received and perceived as motivating, engaging, and supportive of key professional skills. This approach represents a promising complement to traditional medical education, although further controlled studies are required to confirm objective competency gains.

## Introduction

Today’s students are digital natives, living in an always-connected era with instant access to information. This shift has altered their beliefs about learning and their behavior when interacting with educational materials, peers and teachers, especially in digital contexts. Universities will need to recognize that shift in their students, especially in medical education.

On the other hand, the educational community is becoming increasingly aware of how crucial an active learning approach is. This method allows students to transition from being passive recipients of information to taking a more engaged role in their own learning journey (1). Bonwell and Elison describe active learning as “anything that involves students doing things and thinking about them” (2).

This transformation in medical education goes hand in hand with the advance and impact of technology in all areas of society, including medical education. This fosters teamwork and communication skills that are essential in healthcare settings. Moreover, technology encourages interdisciplinary learning, as students from different fields can easily participate in joint training sessions, enhancing their ability to work in diverse teams (3).

Another resource that joins technology and is also part of it is the use of audiovisual media and social networks. Social networks are a perfect example of this, as they blend information and education with entertainment through various formats such as texts, images, videos, and audio. This approach not only motivates students but also helps them feel more involved in the learning process, encourages collaboration, and supports self-directed learning (4). TikTok stands out with its remarkable educational potential, boosting student motivation and fostering active learning (5, 6). Short-form video platforms such as TikTok have specific features that justify their use in medical education. Their strong visual appeal allows complex concepts to be represented in a dynamic and engaging way, while the brevity of the format facilitates the synthesis of information and the delivery of clear key messages. In addition, their cultural affinity with digital-native students and the ease of access via mobile devices promote acceptance and inclusivity. These characteristics make TikTok a particularly suitable tool for fostering motivation, active participation, and the development of communication skills that are essential in medical training. However, in the realm of higher education, particularly among Health Sciences students, research on this topic is still quite limited. While we have found encouraging results in studies involving Nursing and Psychology students (7, 8), there is a significant gap in educational projects that use TikTok for Medical undergraduates. This is a pressing need for research that delves into how this platform can motivate students and be effectively used in this context.

In previous research, the authors examined the use of a short-form video as a learning tool combined with traditional teaching approaches, demonstrating that it can enhance students’ academic performance (9). However, we did not assess how students felt about it or how the use of this tool influenced behavioral patterns such as teamwork collaboration or interaction with digital resources.

This study aimed to analyze the characteristics of student-created videos created by medical students in the field of Radiation Oncology, to evaluate students’ opinions and motivations regarding the effectiveness of short form videos as an active learning tool, and to assess whether this approach improves students’ perception of their mastery of key competencies such as communication skills, time management, and teamwork—all essential for effective medical practice.

## Methods

A cross-sectional educational study was conducted involving 166 students from the Faculty of Medicine at the University of Malaga. These students were enrolled in the Radiation Oncology course during the academic year. The Radiotherapy students were taught with lectures and hospital practices (19 and 10 h, respectively). To improve the teaching/learning process, they were asked to work collaboratively using an additional tool: the production of a short video using the social network TikTok or by recording it locally, on a topic taught in the course to serve as a reinforcement of the knowledge to be acquired (Supplementary Table 1). The formation of working groups and the assignment of topics was carried out randomly.

Participation was voluntary. Students unwilling to create or upload TikTok videos could complete an equivalent alternative task (e.g., a short-written assignment/presentation on the same topic) worth the same 10% of the final grade, communicated in advance through Moodle.

The inclusion criteria were students enrolled in the course and voluntary participation in the activity.

The exclusion criterion was refusal of students to participate in the activity.

**Figure 1:**
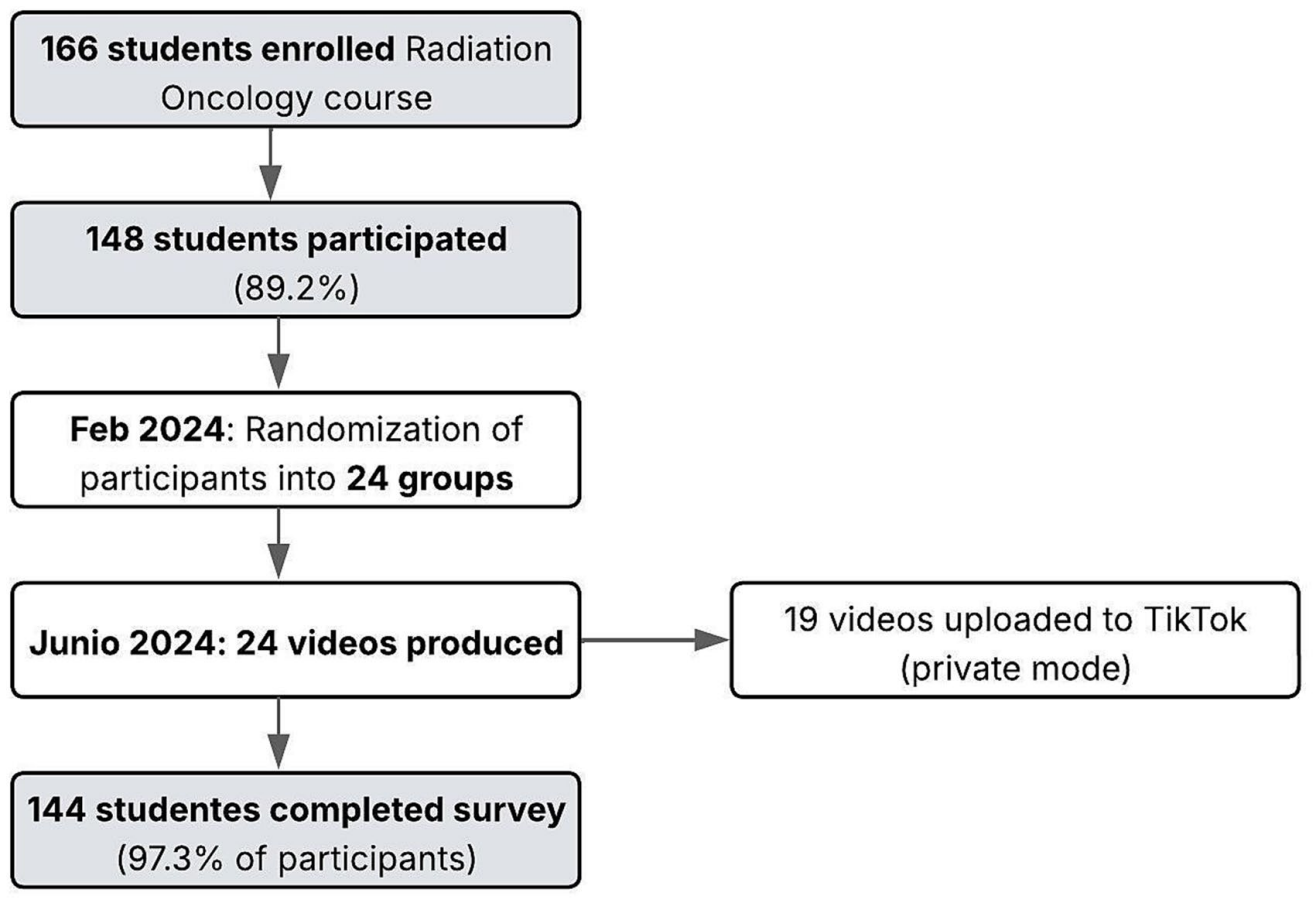
Workflow diagram.

The term “student-created video” has been identified in the literature as student-designed digital media (10). Students were encouraged to use their own notes, online resources from the class, and other reliable materials from the Internet—like images, animations, and video clips—to prepare their videos. None of the students had any experience with professional filming, and they did not have any supervision from the teachers, except for answering any questions that came up. They also did not receive any specific instructions, apart from the guideline that their videos should be no longer than 10 min, which is the current limit for TikTok uploads (11). Of the 166 students enrolled in the Radiation Oncology course, the 148 students who participated inthe activity were randomly divided into groups of six. The activity lasted from February to May and had to be completed by early June, as the evaluation would take place at the end of that month, as detailed in the workflow diagram (Figure 1).

### Ethical and legal considerations

This study involved human participants (undergraduate medical students). The protocol was approved by the Research Ethics Committee of the University of Málaga in year 2024 (CEUMA; approval 137/202/2024). Before participation, all students received written information about the study aims, procedures, potential risks and benefits, and data handling. Participation in the survey and the activity-related data analysis was voluntary and based on written informed consent. To minimize undue influence given the instructor–student relationship, consent and data collection were handled outside of grading procedures, responses were anonymous, and no identifiable information was recorded. Students could complete the assignment without creating a TikTok account and submit their videos via Moodle or local file upload with private sharing settings when applicable. No personal data beyond the video file was collected by the research team.

### Analysis of the characteristics of the video

Among the characteristics of the videos, the following were analysed: the duration of the videos, the audiovisual presentation formats used for their design, and whether they were uploaded to TikTok.

The student videos were evaluated using a rubric developed by the teaching team and previously explained to the students. Two instructors graded the videos simultaneously and jointly, discussing each case in real time until reaching a consensual score. The evaluation assessed creativity, content, dissemination capacity, reliability of the scientific content, and compliance with the presentation time (Supplementary Table 2).

### Assessment instrument

To evaluate students’ opinions and motivations regarding the effectiveness of short-form videos, as well as to assess whether this approach enhanced their perceived mastery of key competencies, we employed a questionnaire originally developed in Spanish by Yélamos-Guerra et al. for foreign language students and validated in a population comparable to our sample (5). For the present study, the instrument was reviewed by a panel of medical education experts to ensure its content relevance.

The final instrument comprised 17 items: 15 closed questions and 2 open-ended questions (Supplementary Table 3). The closed items were grouped into three subscales: Teaching methodology (5 Likert-type and 1 binary items), Perceived usefulness (4 Likert-type and 2 binary items), and Project implementation (3 items including binary and categorical formats). The two open-ended questions explored advantages and suggestions. The survey was created on the University’s Moodle platform and configured so that students who voluntarily chose to complete it were required to answer each question before moving on to the next, ensuring that no missing data could occur in the complete questionnaires.

Reliability was assessed only for the Likert-type subscales, using ordinal alpha coefficients based on polychoric correlations. Reliability estimates were not applied to binary or nominal items.

### Statistical analysis

This study employed a mixed-methods approach, integrating both quantitative and qualitative analyses.

Correlations among Likert-type items were estimated with Spearman’s *ρ*; polychoric correlations were used as robustness checks. Binary–ordinal associations were examined with Mann–Whitney U (reporting Cliff’s *Δ*), and binary–binary with χ^2^ (reporting odds ratios). Holm’s correction was applied to control for multiple testing. Statistical significance was set at *α* = 0.05, and effect sizes with 95% confidence intervals were reported. Analyses were conducted in IBM SPSS v25.1.

Open-ended responses were analysed qualitatively through an inductive thematic approach. Two researchers independently coded the data and reached agreement by consensus. Intercoder reliability was calculated with Krippendorff’s *α* (nominal) to ensure consistency.

## Results

### Analysis of the video characteristics

Of the 166 students enrolled in the Radiation Oncology course, 148 (89.2%) participated in the creation of a video for TikTok.

Twenty-four educational videos were created in total. Some students decided to use free video editing programs like iMovie® or Windows MovieMaker® for their projects, while others recorded live, unedited videos using their cell phones.

The students uploaded 19 videos to the platform (79.2%). Most of the videos (*n* = 20, 83.3%) used digital images or icons and 45.8% (*n* = 11) were accompanied by music.

The videos averaged about 2.97 min, with the shortest clocking in at 1.32 min and the longest stretching to 7.21 min.

Regarding the audiovisual representation formats used, half of the groups (*n* = 12; 50%) used role-playing to communicate their content, where some played the role of the physician and others the patient or caregiver. Nine of the groups (37.5%) acted in an oral presentation format simulating a master’s class with audiovisual support and characterised with a doctor’s gown. Finally, 12.5% (*n* = 3) performed animated audiovisual resources.

### Student opinion and motivation

**Figure 2:**
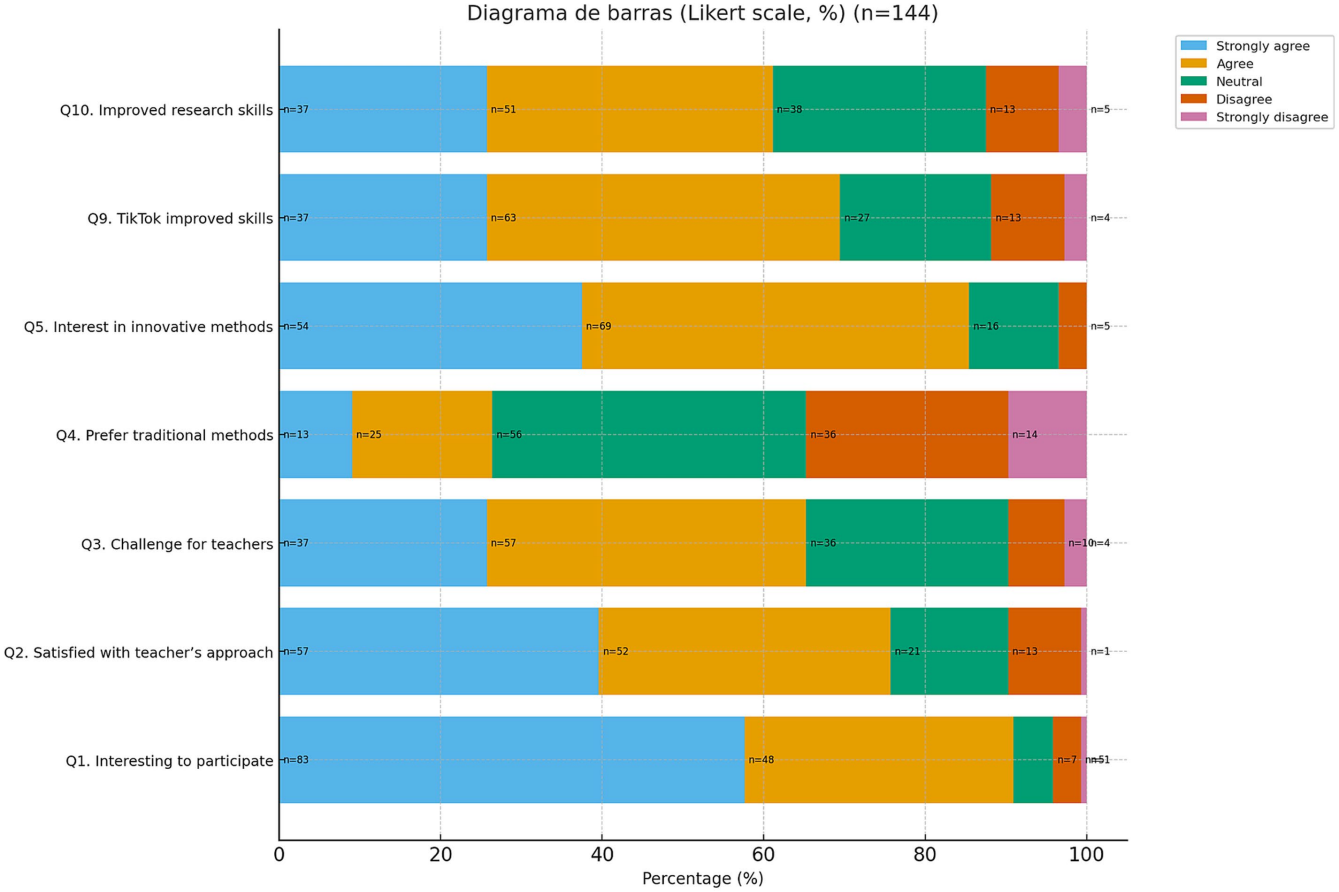
Students’ perception of teaching methodology and perceived usefulness. Responses to the Likert-scale items of the questionnaire, concerning teaching methodology (items 1–5) and perceived usefulness (items 9–10).

A total of 97.3% (*n* = 144) of the students who created the videos completed the survey prior to the final examination. Of these, 91% expressed enthusiasm for innovative methodologies and 76% reported satisfaction with the teaching approach. In addition, 86% reported that the activity helped them internalize content, 87% that it improved memory retention, and 61% that it enhanced research skills. Furthermore, 82% considered it useful for studying Radiotherapy and 73% found it useful for other subjects (Figure 2).

The most frequently reported difficulties were editing/production (46%), organizational demands (40.3%), and technical problems (13.9%). The most enjoyable aspect was teamwork (57%), followed by creativity and communication.

**Figure 3:**
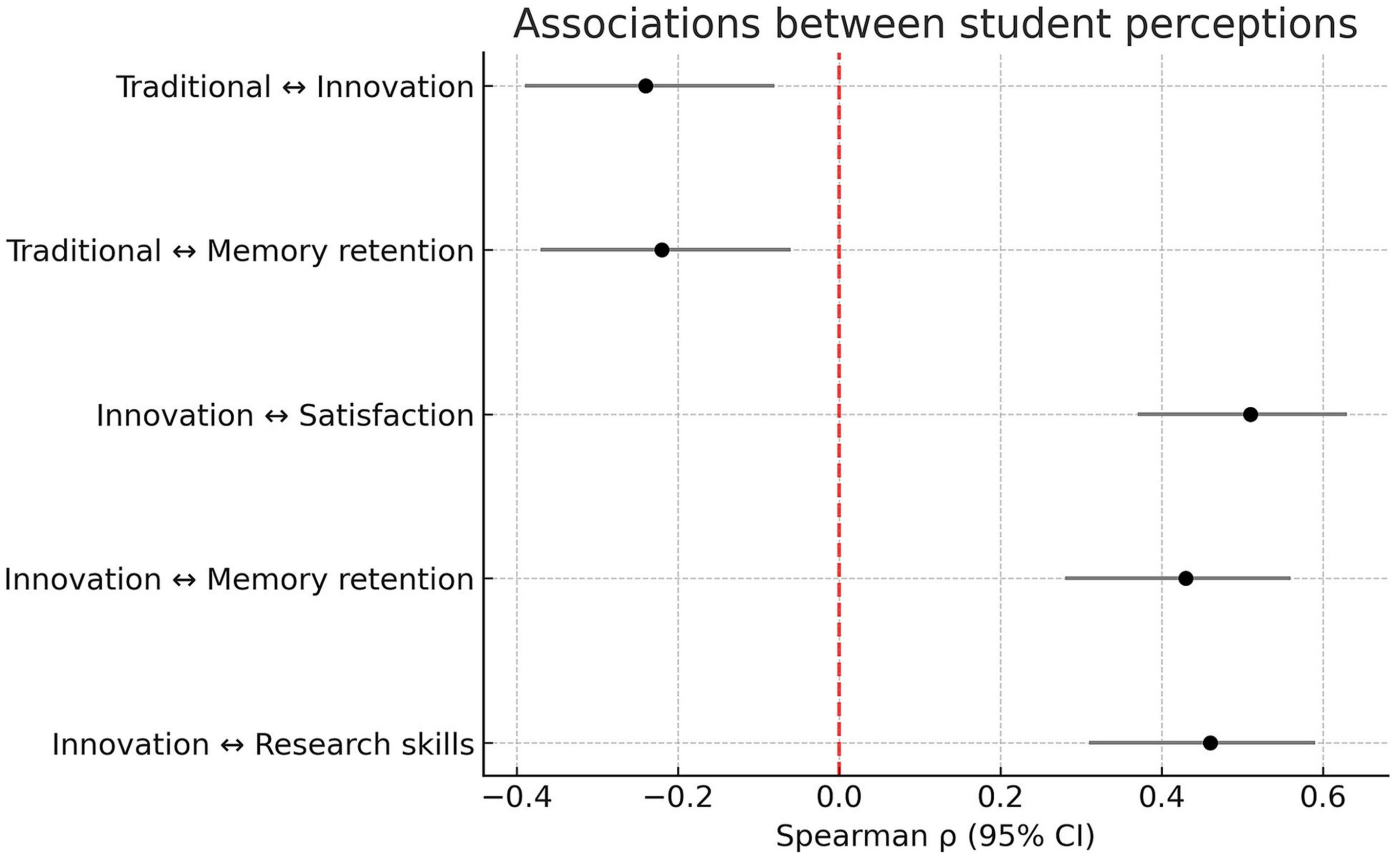
Associations between student perceptions. Forest plot of Spearman correlations (ρ) with 95% confidence intervals between key student perceptions. Correlations are adjusted for multiple comparisons (Holm’s correction). Negative values indicate inverse associations.

When analyzing correlations between questionnaire items, significant positive associations were identified between students’ interest in innovative methodologies and several outcomes, including satisfaction with the methodology (*ρ* = 0.51, 95% CI: 0.37–0.63, Holm-adjusted *p* < 0.001), memory retention (*ρ* = 0.43, 95% CI: 0.28–0.56, p < 0.001), and research skills (*ρ* = 0.46, 95% CI: 0.31–0.59, *p* < 0.001). In contrast, preference for traditional methods was negatively associated with interest in innovation (*ρ* = −0.24, 95% CI: −0.39 to −0.08, *p* = 0.004) and with memory retention (*ρ* = −0.22, 95% CI: −0.37 to −0.06, *p* = 0.008) (Figure 3).

### Advantages and disadvantages

**Figure 4:**
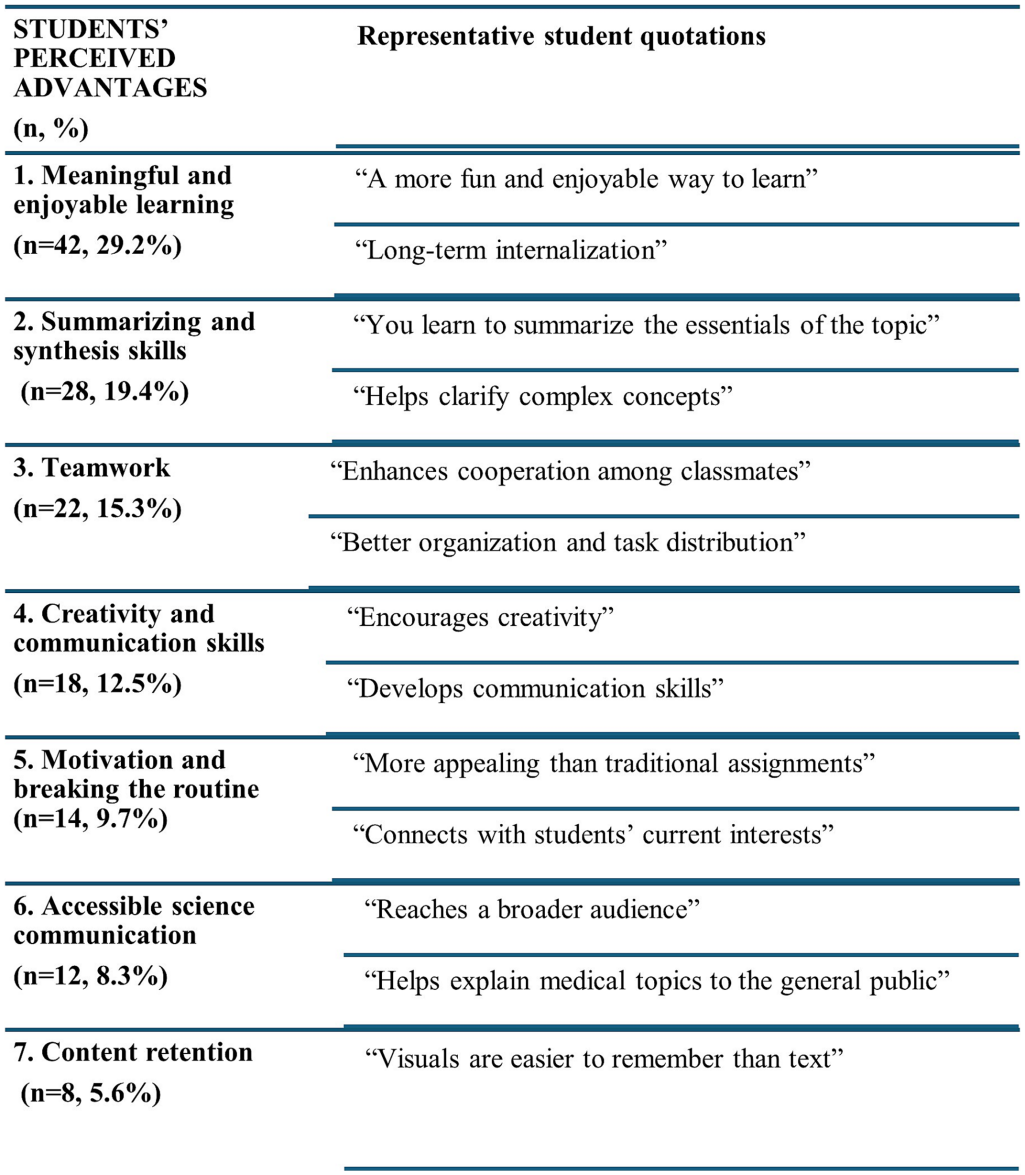
Students’ perceived advantages of the TikTok activity. The main benefits reported by students are presented, with representative quotations included to illustrate each category.

**Figure 5:**
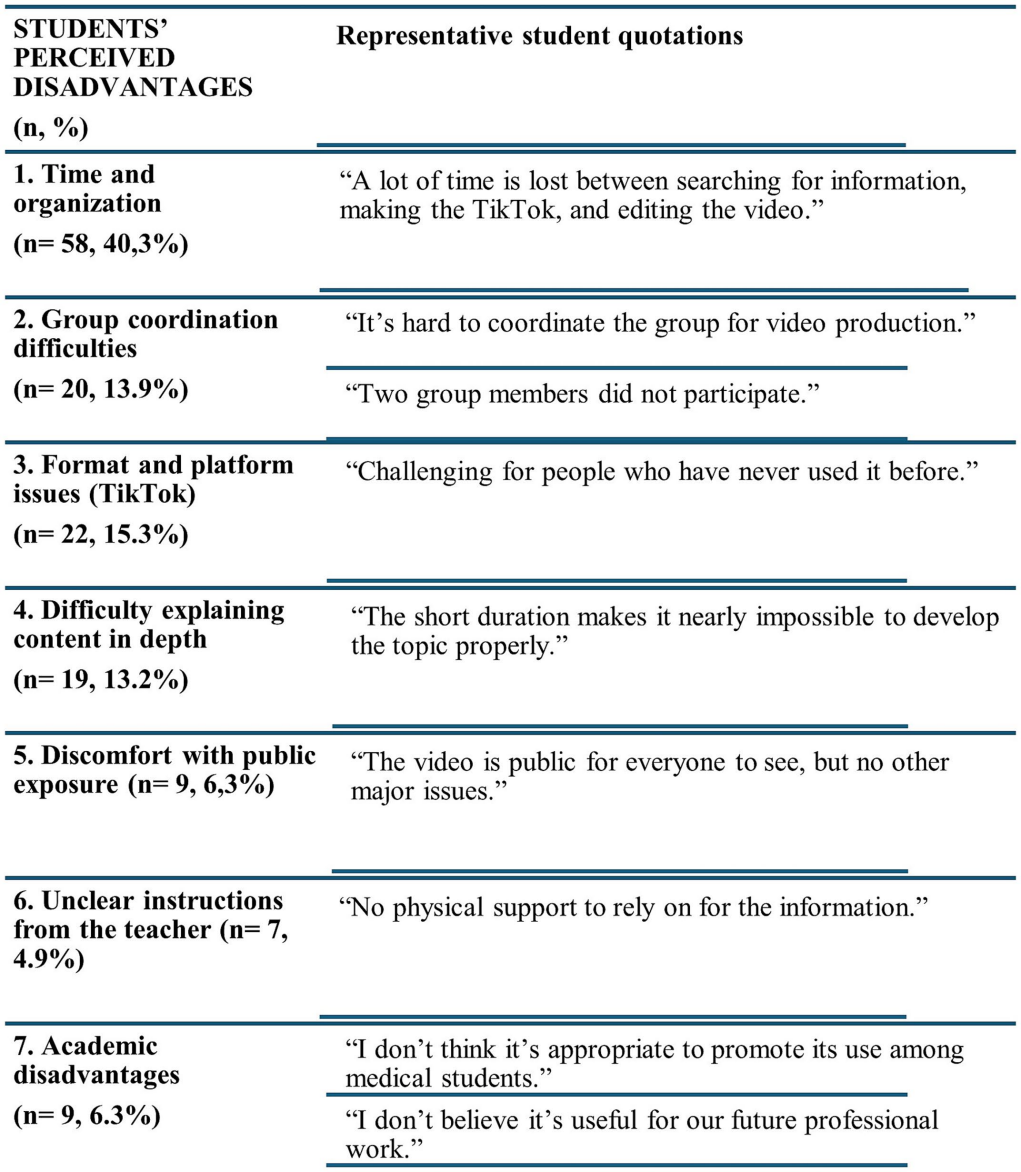
Students’ perceived disadvantages of the TikTok activity. Students’ main reported disadvantages are presented, with illustrative quotations provided for each category.

One of the objectives of this study was to evaluate whether this approach improves students’ perception of their mastery of key competencies for medical practice such as communication skills, time management, and teamwork. In line with this aim, the benefits reported by students were categorized into seven key areas (Figure 4), with representative quotes included to illustrate each category. The most frequently mentioned advantages were that creating the videos made learning more engaging (29.7%) and enhanced students’ ability to convey complex information (19.4%). Other reported benefits included improved teamwork, the development of creativity and communication skills, the ability to communicate scientific content to a broader audience, and better knowledge retention supported by visual aids. Conversely, students also identified disadvantages (Figure 5), particularly the time required for collaborative work and the organizational demands it entailed (40.3%), followed by technical problems with the TikTok platform (13.9%) and limitations due to video length (13.2%). A smaller proportion (6.3%) expressed academic concerns, noting that they did not perceive the activity as useful for their future professional practice.

#### Reliability

As described in the Methods section, open-ended responses were analyzed qualitatively and intercoder agreement was assessed using Krippendorff’s *α* (nominal). Prior to consensus, agreement was substantial (*α* = 0.78), supporting the credibility of the thematic coding.

## Discussion

Contemporary university students have been educated in an environment permeated by technology, within a context in which innovative digital tools are being progressively integrated into educational processes. Among these resources, social media assumes particular significance, as it has demonstrated substantial benefits for collaborative learning (12). It also plays a pivotal role in supporting active learning methodologies (13) and in fostering students’ capacity for self-regulated learning (14). Moreover, the future of medical education is being reshaped by advances in information and communication technologies, underscoring the need to critically re-examine the traditional classroom model. Increased attention should be directed toward these ongoing transformations and toward understanding how medical students respond to and perceive the use of such tools.

### Current results and future of teaching

Students perceived the creation of short-form educational videos as engaging and motivating, reporting benefits in memory retention, internalization of content, teamwork and communication. These findings are consistent with active learning theories and suggest that the traditional classroom model should be re-evaluated considering technological advances that increasingly shape medical education. Similar to Ashraf et al. (3), who emphasized the importance of self-regulation in technology-mediated contexts, our study showed that students took an active role in organizing and presenting knowledge.

From a pedagogical perspective, several educational theories support the integration of these approaches in training environments: constructivism, known for its participatory approach; communication theory, which focuses on the motivation generated by digital interactions (15); and experiential learning, where hands-on experience becomes the main force behind learning (16).

The use of resources such as TikTok can be seen as an innovative instructional method that complements traditional teaching approaches in medicine, such as lectures and clinical practice. By requiring students to select, organise and represent information in a short and accessible audio-visual format, the activity promoted active, self-regulated learning. This process fosters metacognition and demonstrates behaviors typical of self-regulated learning. According to education specialists, fostering self-regulated learning is essential for the education of the future (17).

### Transferability to other subjects

Most students (73%) reported that the activity would be useful beyond Radiotherapy, which supports its scalability to other areas of medical education. This agrees with Poza-Méndez et al. (7), who found that video-based activities favoured the acquisition of competencies in the subject Childhood and Adolescent Nursing. Video-based activities have also yielded remarkable results through a microlearning format when the videos were created by the instructor, as in the work of Conde-Caballero (18) across several modules of the Nursing degree program.

In this way, short videos in TikTok format are not only transferable to other medical courses and to degree programs in the Health Sciences, but can also be developed in various ways, since it has been observed—just as in our study—that students emphasize the motivational impact of integrating audiovisual tools into the curriculum (7, 18).

### Clinical relevance and data protection

As Sadiq et al. suggest, technology-mediated learning requires healthcare professionals to be prepared to integrate digital tools into their teaching practice. By directly experimenting with platforms such as TikTok in an academic setting, medical students not only acquire scientific knowledge, but also develop the communication, digital and pedagogical skills needed to practice in an increasingly technological clinical environment (19).

The skills most positively evaluated—teamwork, communication, and synthesis—were directly relevant to clinical practice. The way students presented their content (often in the role of doctor or teacher) mirrors professional communication tasks, reinforcing the educational value of such activities.

The time management involved in the production of videos contributes to the development of students’ ability to effectively structure medical information for patient communication. However, a potential limitation lies in the challenge of exploring certain topics in depth when time is constrained, as is the case with the short-format nature of platforms like TikTok.

The most enjoyable aspect about the development of the activity for the students was teamwork, since interaction with peers generates the collective construction of knowledge and enriches their medical training (20, 21).

Health professionals have increasingly recognized the power of social networks in sharing quality health information (22, 23). As a result, there is a proposal to incorporate their management into educational curricula, fostering e-professionalism and responsible usage in future job roles (24). Despite the observed educational benefits, it is essential to acknowledge the potential risks associated with the excessive use of these platforms in academic contexts. Prolonged exposure to short-form content may contribute to distraction, information overload, and digital dependency, which can negatively impact students’ well-being and cognitive processing. Therefore, the integration of social media into medical education should be guided by ethical principles. Medical educators are in a privileged position to provide their students with guidance on the appropriate use of social networks in relation to professionalism and data protection (25). This also represents an opportunity to introduce future physicians to the principles of e-professionalism, promoting not only digital competence but also the digital ethics required to respect patient confidentiality, avoid misinformation, and understand the public nature of digital platforms.

### Motivation

The strong visual appeal, brevity, cultural affinity with digital-native students, and ease of access via mobile devices likely explain the high acceptance and participation observed. Similar findings were reported by Yélamos-Guerra et al. (5), who observed high levels of motivation with video-based tasks in language students, Cale et al. (26) also showed that the reliability of these resources increases when created or guided by teachers, which supports the importance of educator involvement.

In terms of training tools, students really valued videos for their ability to boost content retention, help synthesise ideas, and apply knowledge through visual aids. This view is supported by the study conducted by Lu and Chan (21), which revealed that videos enhanced the understanding of complex clinical processes, particularly for students who favour visual and auditory learning styles.

It should be noted that 26% of students reported in the survey that they preferred traditional teaching methods, and this was negatively correlated with the ease of recalling what had been learned from the activity. Kolb’s model (16) helps to explain this finding, particularly the ‘assimilationist’ learning style, which emphasizes a sequential approach and the use of resources such as notes or lectures (27), essentially what we understand as traditional methods.

### Limitations and outlook

A limitation of this study includes the lack of specific and validated assessment tools for this type of intervention, as well as the preference of some students not to participate in the TikTok social network, which was managed by allowing them to upload their videos locally through the university’s Moodle platform. Additionally, prior TikTok use by the participating students was not objectively measured, which may have acted as a confounding factor in their perceptions.

Regarding the questionnaire used in our study, content validity was ensured through expert review and internal consistency was confirmed for the Likert-type subscales. However, the instrument was not subjected to full psychometric validation in medical students. Future research should incorporate additional analyses, such as exploratory and confirmatory factor analysis, test–retest reliability, and criterion validity, in order to further strengthen the instrument for application in this context.

The absence of a control group, reliance on post-test data, and limited psychometric validation of the questionnaire prevent conclusions about objective improvements in competencies and indicate that the results should be understood as perceptions. These limitations echo those described in similar studies using TikTok in higher education (7, 18). Future controlled and longitudinal studies are needed to determine whether the perceived benefits of short-form video creation translate into measurable competency gains.

Nevertheless, it is considered a strength that it addresses a topic that has been little explored in medical students, offering promising results.

From a human behavioral perspective, the results suggest that the use of TikTok promotes self-regulated learning behaviors, proactive collaboration and a sense of belonging to the group. These behavioral changes could be analysed in future research using models such as Bandura’s Social Cognitive Theory (28).

Medical schools worldwide are pursuing future-oriented education, but innovation faces barriers due to faculty’s lack of pedagogical training and the psychological and technical burden of digital transformation. While advanced digital tools offer potential, their complexity often hinders adoption. In contrast, simple and accessible technologies can substantially improve teaching effectiveness as well as enthusiasm and good perception on the part of the students. Institutions are supporting faculty through expert guidance, though at high labor costs. Overall, disruptive innovation in medical education is expected in the near future (29).

## Conclusion

The integration of short videos created by students, through the potential use of TikTok in medical education, was positively received by participants, who highlighted both its usefulness and its motivational impact. With appropriate ethical safeguards and robust methodological rigor, social media platforms may become valuable opportunities for educational innovation in medicine. Although the findings point to potential benefits in terms of engagement and the perceived development of competencies relevant to medical practice, controlled studies are needed to confirm whether these benefits translate into objective and measurable competency gains. Nevertheless, the current results suggest that integrating simple, accessible digital tools like short-form videos can bridge the gap between traditional medical education and the digital competencies demanded by future clinical practice.

## Data Availability

The raw data supporting the conclusions of this article will be made available by the authors, without undue reservation.
