# Supplementary material for: Social media integration in medical training: behavioral impact of short-form video creation as an active learning tool
Source: Front Med (Lausanne). 2025 Oct 29;12:1666255. doi: 10.3389/fmed.2025.1666255 (PMC12605502; doi:10.3389/fmed.2025.1666255)
Supplement: Supplementary file 1 [file Table_1.DOCX]

Supplementary Material

| **Supplementary table 1:** Themes of the videos made by the students |  |  |
| --- | --- | --- |

| Nº | Theme | Nº | Theme |
| --- | --- | --- | --- |
| 1 | Diagnosis of gynecological cancer | 13 | Brachytherapy |
| 2 | Cancer treatment | 14 | Predisposing factors of lung cancer |
| 3 | Gynecological cancer treatment | 15 | Role of radiotherapy in urological tumors |
| 4 | Cancer staging | 16 | Cancer prevention |
| 5 | Role of radiotherapy in lung cancer | 17 | Cancer and hyperthermia |
| 6 | Lung cancer treatment | 18 | Predisposing factors of urological cancers |
| 7 | Role of radiotherapy in breast cancer | 19 | Predisposing factors of ENT tumors |
| 8 | Immunity and cancer | 20 | Energy and radiation types used in radiotherapy |
| 9 | Treatment of ENT tumors | 21 | Cancer diagnosis |
| 10 | Oxygen and radiotherapy | 22 | Lung cancer diagnosis |
| 11 | Breast cancer treatment | 23 | Cancer predisposing factors |
| 12 | Radiation and cancer | 24 | Urologic cancer diagnosis |
