# Supplementary material for: Social media integration in medical training: behavioral impact of short-form video creation as an active learning tool
Source: Front Med (Lausanne). 2025 Oct 29;12:1666255. doi: 10.3389/fmed.2025.1666255 (PMC12605502; doi:10.3389/fmed.2025.1666255)
Supplement: Supplementary file 2 [file Table_2.DOCX]

**Supplementary table 2:** Rubric for evaluation of collaborative work with TikTok.

| **Evaluation** | **10** | **9** | **8** | **7** | **6** | **5** | **4** | **3** | **2** | **1** | **0** | **Final**  **ratings** |
| --- | --- | --- | --- | --- | --- | --- | --- | --- | --- | --- | --- | --- |
| **Creativity** | Original work,  without losing  the  teaching/  divulgative objective,  using  innovative  methodology. |  |  | It does not lose its pedagogical/dissemination focus, but it remains confined to mere exposition, lacking original ideas or creative resources. |  |  | Loses  teaching/  dissemination  aspect, reads  information |  |  |  | Non-  educational/  divulgative,  monotonous  transmission  of content |  |
| **Content** | Clear  explanation of  scientific content, well-argued and  well explained. |  |  | Good scientific  content for the  most part, but  not all of it. |  |  | Content  contains errors |  |  |  | The content is  not scientific  and contains  mostly errors |  |
| **Ability to** | Appropriate  use of  terminology,  oral expression  and clarity |  |  | Not all  terminology  and spoken  language is  appropriate. |  |  | In most cases, appropriate terminology is not employed. |  |  |  | Overall, there  is a lack of  focus and  clarity on the  issue. |  |
| **diffuse** |  |  |  |  |  |  |  |  |  |  |  |  |
| **Reliability of**  **scientific**  **content** | Attachment of scientific bibliographical  sources in the  correct form |  |  | Almost all the  content is  bibliographically referenced  and well  documented. |  |  | It uses few  bibliographical  references and  expresses them  poorly. |  |  |  | No bibliographical  references  are provided |  |
| **Compliance**  **with time** | Adapts to time |  |  | Spends little  time or does  not distribute  the content  well within the  agreed  timeframe |  |  | It takes too  much time or  does not  distribute the  content in the  agreed time. |  |  |  | Completely  exceeds of  time, or does  not reach a  minimum |  |
