# Supplementary material for: Social media integration in medical training: behavioral impact of short-form video creation as an active learning tool
Source: Front Med (Lausanne). 2025 Oct 29;12:1666255. doi: 10.3389/fmed.2025.1666255 (PMC12605502; doi:10.3389/fmed.2025.1666255)
Supplement: Supplementary file 3 [file Table_3.DOCX]

**Supplementary table 3:** Student questionnaire on the implemented teaching methodology and their recorded responses.

| Subscale | Item | Wording (English translation) | Response type |
| --- | --- | --- | --- |
| Teaching methodology | **1** | As a student, do you find it interesting to participate in innovative ways of learning? | 5-point Likert (1 = strongly disagree, 5 = strongly agree) |
|  | **2** | Are you satisfied with the teacher’s methodological approach during the activity? | 5-point Likert |
|  | **3** | Do you think applying this methodology is a challenge for teachers? | 5-point Likert |
|  | **4** | As a student, do you prefer using more traditional teaching methods compared to this activity? | 5-point Likert |
|  | **5** | How do you rate your interest in innovative teaching methods? | 5-point Likert |
|  | **6** | Would you apply this methodology in your classes? | Yes/No |
| Perceived usefulness | **7** | Do you think the video activity significantly helped you internalize the topic content? | 5-point Likert |
|  | **8** | Do you think this activity helps you remember what you've learned? | 5-point Likert |
|  | **9** | Do you think this activity helped you improve your research skills? | 5-point Likert |
|  | **10** | Do you think this activity helped you improve your research skills on a specific topic? | 5-point Likert |
|  | **11** | Do you consider this a useful activity for studying Radiotherapy? | Yes/No |
|  | **12** | Do you consider this a useful activity for studying other subjects? | Yes/No |
| Project implementation | **13** | In your opinion, what was the most difficult aspect of producing the TikTok activity? | Multiple choice (video editing, production, platform use, etc.) |
|  | **14** | In your opinion, what was the easiest and most enjoyable aspect of the TikTok activity? | Multiple choice (teamwork, creativity, etc.) |
|  | **15** | After your experience, would you repeat the activity? | Yes/No |
| Open-ended questions | **16** | Advantages of TikTok implementation | Free text |
|  | **17** | Disadvantages of TikTok implementation | Free text |
